# Believing is seeing: A Buddhist theory of creditions

**DOI:** 10.3389/fpsyg.2022.938731

**Published:** 2022-08-03

**Authors:** Jed Forman

**Affiliations:** Center for Buddhist Studies, University of California, Berkeley, Berkeley, CA, United States

**Keywords:** perception, epistemology, Buddhism, cognitive science, meditation

## Abstract

The creditions model is incredibly powerful at explaining both how beliefs are formed and how they influence our perceptions. The model contains several cognitive loops, where beliefs not only influence conscious interpretations of perceptions downstream but are active in the subconscious construction of perceptions out of sensory information upstream. This paper shows how this model is mirrored in the epistemology of two central Buddhist figures, Dignāga (480–540 CE) and Dharmakı̄rti (c. 550–650 CE). In addition to showing these parallels, the paper also demonstrates that by drawing on Dignāga and Dharmakı̄rti's theory, we can extend the explanatory power of the creditions model. Namely, while creditions explain how beliefs influence both the conscious interpretation and subconscious construction of sensory information, Dignāga and Dharmakı̄rti suggest beliefs can even be *generative* of sensory-like information. I recruit ancient Buddhist texts in conjunction with contemporary cognitive science scholarship to offer a hypothesis for the cognitive mechanisms responsible for this.

## Dignāga and Dharmakı̄rti's epistemology

Dignāga and Dharmakı̄rti's epistemology advocates a sharp divide between perception and inference. On their view, perception is our direct encounter with the world, namely (though not exclusively, as we will see) through the senses. They understand perception as largely causal, with external objects affecting the senses to produce a perception. Inference, on the other hand, uses perceptual information to adduce non-perceptual facts.

The classic example of inference is that of fire from smoke. Because smoke is necessarily created by fire, the perception of smoke warrants an inference of fire. Thus, even when a fire is occluded from our sight, one is justified in concluding there is a fire present after seeing smoke rising. Dignāga and Dharmakı̄rti argue that these two epistemic instruments (perception and inference) give an exhaustive epistemology, explaining all instances of warranted knowledge.

Dignāga and Dharmakı̄rti's differentiation between perception and inference has led some authors to conclude that their theory is a species of sense-data theory. That is, while we perceive colors, shapes, sounds, or textures, we use this information to infer the presence of common-sense objects and medium-sized dry goods. On this view, one never even directly perceives smoke. Instead, one perceives gray forms that are inferred to be “smoke,” and based on this inference, one further infers fire (Arnold, 2017, para. 24, Arnold, 2019, p. 227–228). If this were Dignāga and Dharmakı̄rti's position, it would pit them close to the philosophy of Alfred J. Ayer, who argued that common sense objects are inferred based upon our perception of sense data (Ayer, 1967, p. 129).

There are some aspects of Dignāga's and Dharmakı̄rti's thought that suggest a sense-data theory. Like Ayer, they do argue that we do not perceive medium-sized dry goods, like tables, chairs, peoples, and trees. They consider such objects to be merely conceptual (*vikalpaka*) constructs, reified “universals” (*sāmānya*). As such, they are the referent objects of inferences. Reality itself, on the other hand, is composed of discrete particles that only last for a moment. These are particulars (*svalakṣaṇa*). On this theory, we could think of reality like a buzzing soup of static and white noise. Our tendency to construe enduring, extended objects out of this soup is like a case of ongoing apophenia, the recognition of patterns in otherwise random data.

However, Dignāga and Dharmakı̄rti's theory of conceptualization is distinct from apophenia in an important regard. Unlike apophenia, concepts have pragmatic utility (*arthakriyā*). Dharmakı̄rti gives an analogy to a jewel to make this point. Two people see some shimmering light, and both think that it is a jewel reflecting light. Both cognitions are erroneous (*bhrānti*), since (according to Buddhists) no universal “jewel” inheres in the world. Nevertheless, in one case the light is produced by a lamp and in the other by a group of particulars that collectively have the qualities we would expect of a jewel. In the latter case, then, the cognition is informative (*sam̱vāda*) despite being erroneous, since we can use that cognition to reach particulars that behave in the way we expect of a jewel, even if no jewel is there *really* (Miyasaka, 1972, 2:v.3.57-8; Devendrabuddhi, 1744, F. 145a−146b).

In some ways, this is compatible with Ayer's (perhaps counterintuitive) notion of inference. As a logical positivist, Ayer agrees that the ultimate arbiter of our cognitions is their efficacy, and not whether they represent “real” things. Nevertheless, Dignāga and Dharmakı̄rti's theory of conceptual construction does *not* entail sense data theory. This is because they consider even the apprehension of color to be a conceptual process, a construction of a universal. As Dignāga states, “The apprehension of a color, or the like, [arises] from both the particular, which is ineffable (*avyapadeśya*), and a color, which is a universal” (Hattori, 1968 p. 24 and 81n1.19). In other words, even the recognition of some color involves a constructive process. This follows from Buddhist ontology, since even patches of color (no matter how small) are things that appear to take up time and space.

What, then, *is* perceived according to Dignāga and Dharmakı̄rti? They argue perception perceives particulars. Yet, as Dignāga states, because particulars are completely unique and momentary, they are “ineffable” (*avyapadeśya*). Thus, we cannot say anything about perceptual content, since any such saying is conceptual. This may seem mystical at first. But if we understand perception causally, it becomes less so. “Perception” just means the causal interaction between the senses and the world. It has no content to speak of. Such content only arises to awareness once conceptual processes have done their work (see Sharf, 2018 for details).

This theory comes close to that of another thinker, Charles Peirce.^1^ Like Dignāga and Dharmakı̄rti, Peirce also argues perception is “subconscious” and not operative at the level of awareness. In place of Ayer's inference, he appeals to “abduction” to bridge the divide between perception and our awareness of medium-sized dry goods. Abduction involves pragmatic heuristics that help us navigate our world even though they may misrepresent reality. They are thus “extremely fallible” and updatable as new information arises (Peirce, 1955, p. 304). Like Peirce, Dharmakı̄rti argues that our conceptualizations do not have any necessary authenticity, but are the product of certain “patterns of thought” (ā*hitā vāsanā*) (Gnoli, 1960, 42 ll.13–14). These patterns of thought are preserved or culled to the degree they help us get what we want and avoid what we do not want (Mikogami, 1979).^2^

## Believing is seeing

For anyone familiar with the creditions model of belief formation, Dignāga and Dharmakı̄rti's theory will appear familiar. In the creditions model, awareness of perceptual information only comes at the end of a multistep process. Such information is first parsed through pre-linguistic, “primal” beliefs that are predictive. Like in Dharmakı̄rti's jewel analogy, such beliefs might predict finding a jewel based on the perception of shimmering light. Also like in Dharmakı̄rti's analogy, these beliefs can be refined based on their efficacy. So, if someone sees a shimmer but does not find a jewel, such shimmers will be less likely to produce the assumption of a jewel in the future.

Rüdiger Seitz describes two ways in which these primal beliefs can be updated. The first is through the processing of prediction errors. The person who does not find a jewel updates their valuation processes spontaneously so that they make better predictions. This occurs below the level of awareness. However, these processes can also be updated via conscious awareness. Because beliefs can be semantically encoded into language, we can become aware of them. By reflecting on these beliefs, the brain can affect valuations, changing beliefs and the processing of perceptual information (Seitz et al., 2019; Seitz and Angel, 2020; Seitz, 2022a,b). For example, by reflecting on the irrationality of racist beliefs, one can affect their snap judgements about others.

The creditions model is thus abductive in Peirce's sense and pragmatic in Dharmakı̄rti's. All three models understand cognitive processing to be fallibilist rather than apodictic, updating itself as information arises. However, both Peirce and Seitz present these updates as a transformation of the valuation process. In other words, while the flow of perceptual information stays consistent, it is only how the information is processed that is affected. It is on this point that Dharmakı̄rti offers a variant theory.

On Dharmakı̄rti's theory, perception has greater epistemic weight than inference. This is because all inference is erroneous. To comprehend Buddhist ideas deeply, then, Dharmakı̄rti argues the practitioner must *perceive* these truths in addition to understanding them conceptually. This perceptual understanding is achieved not by sensory perception, but by a special type of perception called “yogic perception” (*yogipratyakṣa*). Dharmakı̄rti explains yogic perception is the product of sustained meditation. He claims that by meditating on some universal, holding it in the mind's eye, the meditator will eventually have “a nonconceptual clear appearance constructed by the power of meditation.” Although this is not an instance of sensory perception, Dharmakı̄rti argues that its clarity is qualitatively indistinguishable from “seeing” something “as if it were right in front of them” (Miyasaka, 1972, 2:v.3.282-4).

Admittedly, it is somewhat unclear what it would be like to “see” an abstract Buddhist concept in such a vivid manner. Nevertheless, Dharmakı̄rti presents an intriguing possibility. If we think of meditation as a type of reflection, Dharmakı̄rti argues that reflective processes do not just affect valuation systems, but perceptual systems as well. In other words, reflection might *generate* perceptual information, not merely *affect* how that information is processed.

In this regard, Dharmakı̄rti offers several analogies to cognitive processes similar to yogic perception. Specifically, he cites hallucinations that are caused by intense emotion, such was when “one is driven crazy by desire, fear, or grief” (Miyasaka, 1972, 2:v.3.282). Dharmakı̄rti's assertion that grief can lead to hallucinations is well documented. Indeed, vivid hallucinations of the deceased are not uncommon during bereavement (Castelnovo et al., 2015). Dharmakı̄rti argues that intense rumination on a loved one eventually spills over into a perceptual event, such that they are no longer just in the mind's eye but seen “as if they were right in front” of the griever. Meditation operates through the same mechanism. By fixating on an idea for a sustained period of time, it will eventually appear clearly and perceptually (Miyasaka, 1972, 2:3.285-6).

## Cognitive underpinnings

Dharmakı̄rti wants to differentiate yogic perception from meditative hallucinations. It is only when the initial meditative idea is “true” that the resultant perception is yogic (Miyasaka, 1972, 2:3.286).^3^ This epistemological issue aside, I want to focus on the mechanisms for how meditation might be generative of novel perceptual content, since the creditions model does not account for such a possibility, nor how it might influence belief formation.

For example, Seitz explains hallucinations as either misinterpretations “triggered by items in the patient's environment” or arising “spontaneously,” perhaps as cognitive misfires (Seitz, 2022a, p. 27). Phillip Gerrans also understands hallucinations as false valuations of perceptual events, “an imaginative state triggered by a sensory or perceptual anomaly” (Gerrans, 2014, p. 137). Seitz's and Gerrans' model would theorize grief hallucinations as the product of over-interpreting sensory information, leading to the sensed presence of a missed loved one. Justin Barrett gives a similar account of the apparition of supernatural agents, where beliefs manipulate the interpretation of sensory information so that bumps and creaks in the night become confirmations of ghosts (Barrett, 2004, chap. 3).

While, indeed, many hallucinations are the product of misinterpretations, others appear too phenomenologically rich to be the result of exaggerations upon sparse perceptual data. For example, consider the following account of a man grieving the loss of his father. The man claims he “was certainly awake” and saw his deceased father in the middle of the night “sitting on the corner of my bed … He was opaque, not ethereal in any way.” What is even more telling about this event is that the griever did not *believe* that he really saw his father. “I do not know whether this was a hallucination or something else, but since I provisionally do not believe in the paranormal, it must have been” (Sacks, 2012, chap. 13). In other words, the hallucination did not appear to be the result of a proclivity to over interpret sensory information to conform with preexisting beliefs. Rather, the hallucination had a perceptual richness *despite* his belief to the contrary. This suggests that something about the reflective process affects not just how perceptual information is interpreted, but can generate perceptual content, even when that content contradicts reflective beliefs.

Although this account is only anecdotal, there is a wealth of evidence that suggests hallucinations can originate from top-down processes, like rumination, in this fashion. To be sure, much, if not the majority, of hallucinatory phenomena is the result of some imbalance between bottom-up perceptual information and top-down predictive coding. Nevertheless, hallucinations can also be the result of top-down processes unilaterally affecting the visual cortex, such as the suppression of sensory signals by the prefrontal cortex (Ranson et al., 2019), coupling between the default mode network (DMN) and the visual cortex (Walpola et al., 2020), and visual cortex activation by higher cortical areas during visualization (Howe and Carter, 2016). The last two examples are especially pertinent to the case of meditation, since what Dharmakı̄rti has in mind is an intense visualization practice—which is either instigated be intense emotion, such as grief, or the result of deliberate cultivation. Several studies reveal that meditation increases DMN-visual-cortex coupling (Faber et al., 2014; Berkovich-Ohana et al., 2016; Fujino et al., 2018; Zhang et al., 2021), which may offer a mechanism of how deliberate meditation induces hallucinations.

Another possible mechanism to explain vivid hallucinations induced by meditation is hypnosis. Some research suggests that hypnosis and meditation create vivid visual experiences through a shared mechanism. Namely, both *downregulate* executive prefrontal systems as well as the DMN (Dietrich and Al-Shawaf, 2018), creating a space within which imaging systems can create vivid representations from the bottom-up, unimpeded by prefrontal regulation (Winkelman, 2017). Even though meditation is highly focused, the high recruitment of attentional systems in both meditation and hypnosis creates hypoactivity in other prefrontal systems, leading to deregulation (Dietrich and Al-Shawaf, 2018). However, recent scholarship has brought this hypofrontality thesis into question (Fingelkurts et al., 2007; Facco, 2021). Thus, other scholarship concludes that hypnosis enhances the vividness of mental imagery top-down via the prefrontal cortex (Sireteanu et al., 2010; Lanfranco et al., 2021). This might explain how images in the mind's eye can become vividly visual *via* deliberate meditative practice.

In sum, this research suggests at least three possible mechanisms through which meditation might produce perceptual content: (1) the coupling of the visual cortex with other cognitive systems, (2) the downregulation of prefrontal systems, letting imagery bubble up from the bottom up, and (3) the creation of vivid imagery from the top down. It is not unlikely that all these procedural alternatives are possible, meaning that visual hallucination is overdetermined by meditative practice. Indeed, there are many different types of meditative practices, each of which may exploit these pathways differently.

Our analysis thus reveals that higher-order cognitive processes, like reflection, might not just transform how perceptual information is processed, but may generate perceptual content itself. In other words, belief may not just manipulate *how* we see but generate *what* we see.

## Conclusion

Dignāga and Dharmakı̄rti's theory thus shares many affinities with the creditions model, particularly concerning how belief formation develops under normal circumstances. Both theories argue that perception is causal and subconscious, that perceptual awareness is highly entangled with beliefs about the world, and that these beliefs are fallible, formed by abductive processes that are patterned by experience.

Dignāga and Dharmakı̄rti, however, present an additional picture of how this processing can flow in special circumstances. That is, beliefs do not just organize perceptual information upstream nor merely interpret that information downstream. In rare cases, beliefs can generate perceptual information itself. Dignāga and Dharmakı̄rti argue that it is only in some cases that this process is epistemic, when these starting beliefs are “true.” But if we bracket epistemology, meditative hallucinations may be instrumental in *belief* formation, regardless of whether those beliefs constitute knowledge. For example, fixation on concepts like “ghost” might not just cause someone to *interpret* perceptual data as ghosts, but *produce* the perception of a ghost, reinforcing their belief in ghosts.

The hypothesis at this point is speculative. Future research could use fMRI imaging to gain a closer look at how meditation affects the visual cortex, and whether that activity is highly correlated with visual hallucinations. Such research should be sensitive to the meditative practices involved, particularly whether they are the type of concentration-demanding practices described by Dharmakı̄rti.

If meditation does prove to be generative of perceptual content in the fashion hypothesized, then it offers another important clue into the phenomenon of belief formation, especially of the religious sort. That is, religious beliefs might not merely arise as ways to make sense of aberrant sensory experiences (as in Seitz's and Gerrans' model), nor do they merely persist as intuitive explanations of our sensory world (Sperber, 1996, p. 98–118). In addition to these modes, beliefs may also generate their own perceptual content in a manner that makes them self-confirming. Tanya Luhrman's work has also explored this possibility (Luhrmann, 2012). If this is true, what remains to be seen is the pervasiveness of these experiences—whether they are only the provenance of elite practitioners engaged in meditative practices, or they are operable even among a wider population and explain the persistence of their religious beliefs.

## Author contributions

The author confirms being the sole contributor of this work and has approved it for publication.

## Funding

This paper is funded by Dr. Rüdiger Seitz, *via* the Volkswagen Foundation, Siemens Healthineers, and the Betz Foundation. Siemens Healthineers were not involved in the study design, collection, analysis, interpretation of data, the writing of this article or the decision to submit it for publication.

## Conflict of interest

The author declares that the research was conducted in the absence of any commercial or financial relationships that could be construed as a potential conflict of interest.

## Publisher's note

All claims expressed in this article are solely those of the authors and do not necessarily represent those of their affiliated organizations, or those of the publisher, the editors and the reviewers. Any product that may be evaluated in this article, or claim that may be made by its manufacturer, is not guaranteed or endorsed by the publisher.

## Footnotes

^1^Dunne (2004, p. 49) also notes parallels between Dharmakı̄rti and Peirce, though he cautions not to overstate the similarities.

^2^In Dharmakı̄rti's epistemology, “conceptual habituation” (*vikalpābhyāsa*) probably comes closest to Peirce's abduction, which describes how repeated experience leads to an automatic cognitive association between the perceptual stimulus and a given concept (Eltschinger, 2014, §1.2; Kellner, 2004, p. 30–31).

^3^There is debate in the secondary literature as to how Dharmakı̄rti make this differentiation. Compare Dunne (2007, p. 515) vs. Eltschinger (2009, 169n1) and Franco (2011, 87 ff.).
